# Impact of the COVID-19 pandemic on growth determinants in premature neonates: observational study in a secondary health service in Carapicuíba, São Paulo (2020–2023)

**DOI:** 10.3389/fped.2024.1431402

**Published:** 2024-12-04

**Authors:** Vanessa Marques Leite Martha, Deborah Cristina Landi Masquio, Luiz Silva dos Santos, Julia Marques Leite Martha, Pedro Marques Leite Martha, Guido de Paula Colares Neto

**Affiliations:** ^1^ Department of Nutrition, Centro Universitário São Camilo, São Paulo, Brazil; ^2^ Department of Psychology, University of São Paulo, São Paulo, Brazil; ^3^ Department of Economics, Insper Institute of Education and Research, São Paulo, Brazil

**Keywords:** prematurity, growth, nutrition, pandemic, COVID-19

## Abstract

**Introduction:**

Prematurity is a factor that contributes to the increase in infant morbidity and mortality and is associated with factors determining child growth, such as nutritional deficits, mainly during the COVID-19 pandemic.

**Objective:**

To evaluate the factors determining the growth of premature neonates treated at a secondary health service during the COVID-19 pandemic.

**Method:**

Observational retrospective and prospective cohort study of premature patients followed at the follow-up clinic in the municipality of Carapicuíba from February 2020 to December 2023. Through a review of medical records, anthropometric data were collected from birth to corrected gestational age, approximately six months, and nutritional and non-nutritional data with direct or indirect influence on growth. Statistical analysis with tests for quantitative and qualitative variables was carried out with the SPSS Statistics software version 27.0 (SPSS et al., USA).

**Result:**

The study analyzed a sample of 302 newborns, predominantly male (51.7%) and classified as moderately preterm (47.4%), with an average gestational age of 32.4 ± 2.8 weeks. Prenatal complications occurred in 59.3% of cases, while neonatal complications, such as extrauterine growth restriction (EUGR, 30.8%) and use of parenteral nutrition (36.1%), were common, with an average hospital stay of 30.2 ± 26.1 days. Most newborns were artificially fed (51%) Moreover, they received multivitamins (71.9%). At six months, the newborns showed healthy growth with an average weight of 6.718.2 ± 1.346.5 g. Multiple linear regression analysis revealed significant associations between EUGR and negative Z scores for weight, length, and head circumference at six months. Complications such as anemia and congenital abnormalities also negatively impacted these scores. Comparatively, the newborns in the group pre-vaccination against COVID-19 had more significant growth at six months and more prevalence of newborns born large for gestational age (LGA), while complications such as gastroesophageal reflux disease and viral bronchiolitis was more common in the post-vaccination group.

**Conclusion:**

During the COVID-19 pandemic, non-nutritional factors, especially EUGR, significantly influenced the anthropometry of premature babies. This condition highlighted the need for more effective therapeutic strategies and public health measures to improve the growth and development of premature infants.

## 1 Introduction

Prematurity, defined as birth before 37 weeks of gestation, is a complex condition, with multifactorial etiology and significant implications for global public health and the burden of chronic non-communicable diseases and disabilities for the child. In Brazil, prematurity is the leading cause of infant death in the first five years of life, with regional variations that reflect socioeconomic inequalities. Thus, the COVID-19 pandemic brought new challenges and impacts on this condition (1–4).

The COVID-19 pandemic declared a public health emergency of international concern by the World Health Organization in January 2020 and by the Ministry of Health of Brazil in February 26, 2020 (5), exacerbated the vulnerabilities of premature babies. According to the COVID-19 Bulletin of the Oswaldo Cruz Foundation (6), although the vaccination campaign, which began in January 17, 2021, reduced cases and deaths between July and November, a new wave of transmission emerged in December, increasing cases, especially among children, who only started to be vaccinated in 2022.

Studies indicate that pregnant women infected with SARS-CoV-2 are at greater risk of premature births (7, 8). Social distancing measures and fear of infection contributed to changes in care and nutrition practices for premature babies, such as the decline in exclusive breastfeeding and the early adoption of complementary feeding, with a possible impact on the growth and development of premature babies (9, 10). During the pandemic, monitoring anthropometric indicators became critical, considering the possible impacts of COVID-19 pandemic, the response measures on access to healthcare and the nutrition of premature babies (5, 7, 8).

This article aims to analyze the nutritional and non-nutritional factors that influenced the anthropometry of premature infants during their postnatal growth before the COVID-19 vaccine and after the vaccination period.

## 2 Methods

This work was submitted to the Research Ethics Committee (CoEP) of Centro Universitário São Camilo and approved under Certificate of Presentation of Ethical Appreciation 72834523.7.0000.0062 and number opinion 6.304.909. Data collection occurred after those responsible agreed with the free and informed consent terms. After acceptance, data were collected from the medical records.

A mixed-method approach was employed in a retrospective and prospective cohort study, integrating quantitative and qualitative methodologies. The study draws on secondary data from medical records of premature infants treated at the follow-up clinic in Carapicuíba, Brazil, from February 26, 2020, to December 30, 2023. Data collection from 2020–2022 was conducted retrospectively from patient medical records, while data from 2023 were gathered prospectively, incorporating information from medical consultations and patient records.

The inclusion criteria were premature infants born at less than 37 weeks of gestational age. They had their first post-discharge consultation before six months of corrected gestational age, with at least one additional consultation between five and seven months. The corrected gestational age was calculated using the following formula: corrected gestational age = chronological age—(40–gestational age at birth) (11), reached 40 weeks. The exclusion criteria included infants with fewer than two outpatient visits in the first six months of life, those who did not return between the fifth and seventh months of corrected gestational age, and those with conditions that hinder standard anthropometric measurements using a horizontal ruler, such as malformations, metabolic disorders, or congenital/skeletal deformities with muscle contractures. Other congenital abnormalities, such as cardiac or pulmonary conditions, were included.

Eligible patients were analyzed in their entirety and divided into two groups: the pre-vaccination group, consisting of those born before the start of COVID-19 vaccination in Brazil (January 17, 2021), and the post-vaccination group, consisting of those born after that date.

In this study, prematurity was categorized into three distinct levels based on gestational age at birth. Extreme prematurity was defined as birth occurring between 22 weeks and 28 weeks incomplete. Moderate prematurity included births between 28 weeks and 34 weeks incomplete, while late prematurity referred to births between 34 weeks and 37 weeks incomplete.

Anthropometric measurements of weight, length, and head circumference were collected at birth, hospital discharge, and around six months of corrected age (between 5 and 7 months). The anthropometric data collection followed the standardization proposed by the Ministry of Health of Brazil (12). The data was evaluated in a Z score of weight for age, length for age, and head circumference for age, as recommended by the Intergrowth-21st growth curves (11) and adjusted WHO curves (13).

Regarding weight adequacy for gestational age according to the Intergrowth- 21st curve for those over 33 weeks and Fenton curve for those under 33 weeks, the newborns were classified as small for their age gestational (SGA) (below the 10th percentile), appropriate for gestational age (AGA) (between the 10th and 90th percentiles) and large for gestational age (LGA) (above the 90th percentile) (14, 15).

It was considered as a dependent variable the variation in the standard deviation (SD) of anthropometric data at birth, at hospital discharge, and 6 (5–7) months of corrected gestational age (64 weeks). It analyzed prenatal determinants (gestational complications, such as preeclampsia, premature rupture of membranes, oligohydramnios, urinary tract infection, gestational diabetes, gestational hemorrhages, cervical insufficiency, alloimmunization, infectious and contagious diseases; health behaviors; maternal and biological factors of the newborn), postnatal- non nutritional determinants (diseases diagnosed during hospitalization in the neonatal intensive care unit—ICU or outpatient follow-up), presence of extrauterine growth restriction (EUGR) at hospital discharge (Z score of weight at hospital discharge less than Z −2) and practices of post-discharge feeding (types of breastfeeding as defined by the Brazilian Ministry of Health and World Health Organization (11, 12, 16).

Data analysis covered quantitative and qualitative methods. The dependent variables were the variation in SD of anthropometric data at birth, hospital discharge, and at six months of corrected gestational age. For the descriptive quantitative variables analysis, measures of central tendency such as mean and median, and dispersion (DP, minimum and maximum values) were used. For categorical variables, measures of frequency (absolute and relative) were implemented. The normal distribution or not of the data was verified using the Kolmogorov-Smirnov test. For group comparison, the Student's *T* test was used for independent samples, and the Mann-Whitney tests were used for non-parametric data. Correlations between variables were performed using the Spearman and Pearson tests according to normality distribution. The Chi-square test was used to verify the magnitude of association between the categorical study variables, considering *p* ≤ 0.05 as significant Multiple linear regression tests were applied to identify factors associated with anthropometry. Analysis of variance (ANOVA) was used to compare groups and within independent groups. Statistical analysis was performed with the SPS Statistics program software version 27.0.

## 3 Results

The sample characteristics of 302 newborns are described in Table 1.

**Table 1:** Neonatal characteristics of the total sample.

Sample characteristics	
Total patients (*n*; %)	302 (100)
Sex	
Male/Female (*n*; %)	156/146 (51.7/48.3)
Classification of prematurity	
Late pre-term (*n*; %)	130 (43)
Moderate preterm (*n*; %)	143 (47.4)
Extreme preterm (*n*; %)	29 (9.6)
Gestational characteristics	179 (59.3)
Prenatal complications (*n*; %)	
Maternal addictions (*n*; %)	21 (7.0)
Twinship (*n*; %)	64 (21.2)
Characteristics at birth	
1st/5th minute Apgar	7.09 ± 1.69/8.64 ± 0.94
Weight at birth	
Extremely low weight (*n*; %)	27 (8.9)
Very low weight (*n*; %)	79 (26.2)
Low weight (*n*; %)	171 (56.6)
Suitability for gestational age	
Small for gestational age (*n*; %)	42 (13.9)
Suitable for gestational age (*n*; %)	257 (85.1)
Large for gestational age (*n*; %)	3 (1)
Neonatal complications	
Length of stay (days)	30.2 ± 26.1
EUGR (*n*; %)	93 (30.8)
Parenteral nutrition (*n*; %)	109 (36.1)
Neonatal sepsis (*n*; %)	102 (33.8)
Perinatal asphyxia (*n*; %)	16 (5.3)
Respiratory distress syndrome (*n*; %)	103 (34.1)
Periventricular hemorrhage (*n*; %)	34 (11.3)
Bronchopulmonary dysplasia (*n*; %)	52 (17.2)
Patent ductus arteriosus (*n*; %)	56 (18.5)
Anemia (*n*; %)	75 (24.8)
Congenital abnormalities (*n*; %)	38 (12.6)
Type of breastfeeding and use of supplements	
Exclusive breastfeeding (*n*; %)	41 (13.6)
Artificial breastfeeding (*n*; %)	154 (51)
Mixed breastfeeding (*n*; %)	89 (29.5)
Multivitamin (*n*; %)	217 (71.9)
Ferrous sulfate (*n*; %)	270 (89.4)
Post-discharge complications	
Post-discharge complications (*n*; %)	95 (31.5)
Gastroesophageal reflux disease (*n*; %)	46 (15.2)
Wheezing infant (*n*; %)	42 (13.9)
Urinary tract infection (*n*; %)	8 (2.6)
Diarrhea (*n*; %)	7 (2.3)

The sample comprises a slightly higher proportion of male newborns (51.7%), the majority classified as moderately preterm (47.4%) with a mean gestational age of 32.4 ± 2.8 weeks. Prenatal complications were frequent (59.3%), and the most prevalent neonatal complications were the use of parenteral nutrition (36.1%), neonatal sepsis (33.8%), respiratory distress syndrome (RDS) (34.1%) and EUGR (30.8%), and the average length of stay was 30.2 ± 26.1 days. Regarding the type of breastfeeding and the use of supplements, most received artificial breastfeeding (51%) and multivitamins (71.9%).

Preterm infants were born with an average weight of 1.772.5 ± 560.5 g (Z score of weight −0.33 ± 0.96) with a greater prevalence of newborns with low birth weight (56.6%) and classified as AGA (85.1%). Most patients had adequate length with a mean of 41.3 ± 4.2 cm (Z score of length −0.62 ± 1.18) and head circumference of 29.3 ± 2.9 cm (Z score of head circumference −0.33 ± 1.23) with 10.3% of newborns with microcephaly at birth—Table 2.

**Table 2:** Anthropometric evolution of the total sample at birth and at 6 months of corrected gestational age.

	At birth	6 months
Anthropometry		
Gestational age (s)	32.4 ± 2.8	62.6 ± 6.8
Weight (g)	1,772.5 ± 560.5	6,718.2 ± 1,346.5
Z score of weight	−0.33 ± 0.96	−0.61 ± 1.27
Length (cm)	41.3 ± 4.2	63.4 ± 4.7
Z score of length	−0.62 ± 1.18	−0.66 ± 1.49
Head circumference (cm)	29.3 ± 2.9	41.8 ± 2.5
Z score of head circumference	−0.33 ± 1.23	−0.23 ± 1.52
Height classification		
Short stature (*n*; %)	41 (13.6)	50 (16.6)
Adequate height (*n*; %)	257 (85.1)	243 (80.5)
High stature (*n*; %)	4 (1.3)	9 (3)
Head circumference classification		
Microcephaly (*n*; %)	31 (10.3)	28 (9.3)
Macrocephaly (*n*; %)	8 (2.6)	14 (4.6)
Anthropometric evolution		
Δ Z score of weight	–	0.97 ± 1.15
Δ Z score of length	–	−0.03 ± 1.44
Δ Z score of head circumference	–	0.09 ± 1.52

Categorical variables presented in absolute values (*n*) and relative percentage (%); Numeric variables presented as mean ± standard deviation; *n*, number; s, weeks; g, grams; Δ, variation.

At six months after birth, corrected gestational age was 62.6 ± 6.8 weeks, and mean weight was 6.718.2 ± 1.346.5 g (Z score of weight −0.61 ± 1.27). The weight gain Z score from birth to 6 months was −0.61 ± 1.27, which resulted in eutrophic in most infants, but there was a higher prevalence of high BMI for age (10.9%) than thinness or severe thinness (7%). The mean length was 63.4 ± 4.7 cm (Z score of length −0.66 ± 1.49) with a mean gain in Z score of length from birth to 6 months of −0.66 ± 1.49, which resulted in a slightly increased prevalence of short stature (16.6%) at six months compared to birth. The average head circumference was 41.8 ± 2.5 cm (Z score of head circumference −0.23 ± 1.52), and the average gain in Z score from birth to 6 months was 0.97 ± 1.15 with a slight reduction in the prevalence of microcephaly (9.3%) and an increase in macrocephaly (4.6%)—Table 2.

In the multiple linear regression analysis applied to the sample, it was observed that the EUGR presented a substantial negative association with Z score of weight (*β* = −0.331, *p* = 0.000), length (*β* = −0.341, *p* = 0.000), and head circumference (*β* = −0.213, *p* = 0.001) of premature babies at six months. Congenital abnormalities also demonstrated adverse effects in the Z score of weight (*β* = −0.255, *p* = 0.000) and head circumference (*β* = −0.178, *p* = 0.001). At the same time, anemia was negatively associated with Z score of weight at six months (*β* = −0.193, *p* = 0.009) and patent ductus arteriosus on the Z score of length (*β* = - 0.135, *p* = 0.020).On the other hand, neonatal sepsis positively influenced both the Z score of weight (*β* = 0.164, *p* = 0.015) and length (*β* = 0.149, *p* = 0.036) (data not presented in tables).

In the analysis of variance related to the type of breastfeeding used in the total sample, it was found that breastfeeding impacted the Z score of weight and head circumference (*p* = 0.01 and *p* = 0.04 respectively). Infants submitted to artificial breastfeeding with added carbohydrates and mixed breastfeeding presented higher means in Z scores for weight and head circumference (data not presented in the table).

The comparison between the pre and post-vaccination groups against COVID-19 revealed homogeneity in most variables, but there was a higher corrected gestational age at six months and a higher prevalence of LGA newborns in the pre-vaccination group (*p* = 0.02). Regarding prematurity, the post-vaccination group tended to have a higher prevalence of moderate (*p* = 0.09) and late (*p* = 0.02) prematurity compared to the pre-vaccination group. As to complications after hospital discharge, gastroesophageal reflux disease (*p* = 0.04), wheezing (*p* = 0.01), and viral bronchiolitis (*p* = 0.01) were more prevalent in the post-vaccination group—Table 3.

**Table 3:** Comparison of characteristics between groups according to birth pre and post-vaccination period against COVID-19.

Sample characteristics	Group	Group	*p* *
	Pre-Vaccination	Post-Vaccination	
Total patients (*n*; %)	86 (100)	216 (100)	
Sex			
Male/Female (*n*; %)	43 (50)/43 (50)	113 (52.3)/103 (47.7)	
Classification of prematurity			
Late pre-term (*n*; %)	46 (53.5)	84 (38.9)	0.02*
Moderate preterm (*n*; %)	34 (39.5)	109 (50.5)	0.09
Extreme preterm (*n*; %)	6 (7)	23 (10.6)	0.39
Gestational characteristics			
Prenatal complications (*n*; %)	46 (53.5)	133 (61.6)	0.24
Maternal addictions (*n*; %)	5 (5.8)	16 (7.4)	0.80
Twinning (*n*; %)	14 (16.3)	50 (23.1)	0.21
Characteristics at birth			
1st/5th minute Apgar	7.2 ± 1.8/8.7 ± 1.1	7.0 ± 1.6/8.6 ± 0.8	0.42/0.27
Birth weight			
Extremely low weight (*n*; %)	7 (8.1)	20 (9.3)	0.82
Very low weight (*n*; %)	16 (18.6)	63 (29.2)	0.06
Low weight (*n*; %)	52 (60.5)	119 (55.1)	0.44
Suitability for gestational age			
Small for gestational age (*n*; %)	11 (12.8)	31 (14.4)	0.85
Suitable for gestational age (*n*; %)	72 (83.7)	185 (85.6)	0.72
Large for gestational age (*n*; %)	3 (3.5)	0 (0)	0.02*
Neonatal complications			
Length of stay (days)	28.7 ± 26.8	30.7 ± 25.9	0.54
EUGR (*n*; %)	23 (26.7)	70 (32.4)	0.40
Parenteral nutrition (n;%)	26 (30.2)	83 (38.4)	0.19
Neonatal sepsis (*n*; %)	34 (39.5)	68 (31.5)	0.22
Perinatal asphyxia (*n*; %)	5 (5.8)	11 (5.1)	0.78
Respiratory distress syndrome (*n*; %)	25 (29.1)	78 (36.1)	0.28
Periventricular hemorrhage (*n*; %)	9 (10.5)	25 (11.6)	0.84
Bronchopulmonary dysplasia (*n*; %)	14 (16.3)	38 (17.6)	0.86
Patent ductus arteriosus (*n*; %)	9 (10.5)	47 (21.8)	0.02*
Anemia (*n*; %)	17 (19.8)	58 (26.9)	0.24
Type of breastfeeding and use of supplements			
Exclusive breastfeeding (*n*; %)	11 (12.8)	30 (13.9)	0.85
Artificial breastfeeding (*n*; %)	45 (52.3)	109 (50.7)	0.80
Mixed breastfeeding (*n*; %)	25 (29.1)	64 (29.6)	1.00
Multivitamin (*n*; %)	65 (75.6)	152 (70.4)	0.39
Ferrous sulfate (*n*; %)	77 (88.5)	193 (89.4)	1.00
Post-discharge complications			
General complications (*n*; %)	24 (27.9)	71 (32.9)	0.49
Gastroesophageal reflux disease (*n*; %)	19 (22.1)	27 (12.5)	0.04*
Wheezing (*n*; %)	5 (5.8)	37 (17.1)	0.01*
Urinary tract infection (*n*; %)	3 (3.5)	5 (2.3)	0.69
Diarrhea (*n*; %)	2 (2.3)	5 (2.3)	0.69
Viral bronchiolitis (*n*; %)	14 (16.3)	66 (30.6)	0.01*
Congenital abnormalities (*n*; %)	15 (17.4)	23 (10.6)	0.12
Anthropometric recovery in 6 months			
Weight (*n*; %)	75 (87.2)	185 (85.6)	0.85
Length (*n*; %)	75 (87.2)	176 (81.5)	0.30
Weight and length (*n*; %)	71 (82.6)	173 (80.1)	0.74

Categorical variables presented in absolute values (*n*) and relative percentage (%); Numeric variables presented as mean ± standard deviation; *n*: number; EUGR: extrauterine growth retardation.

* *T* student test for numerical variables and Chi-square test for categorical variables.

**Figure 1:**
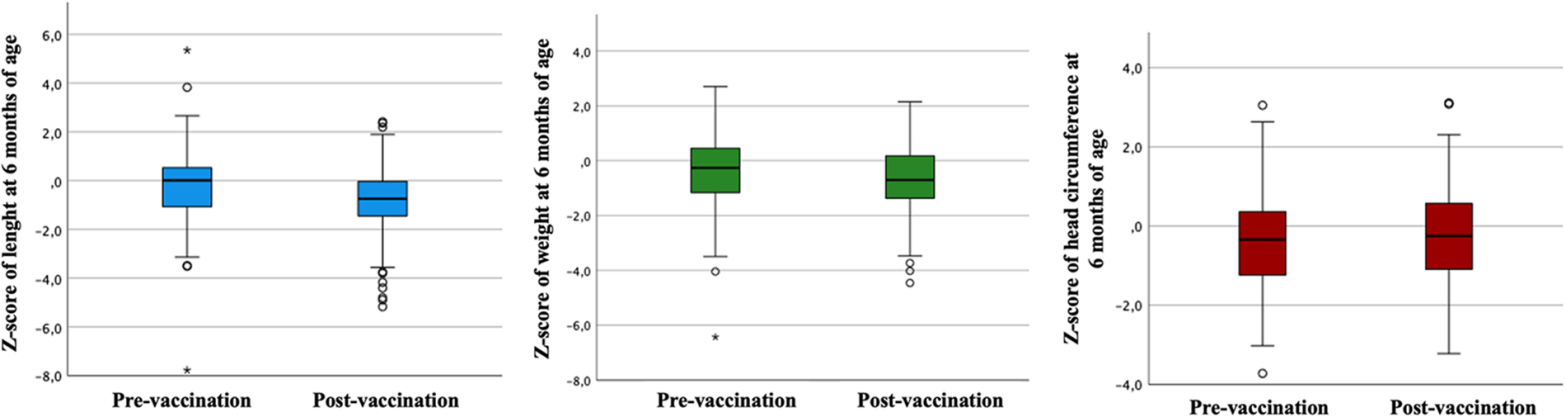
Comparison of Z-scores for length, weight, and head circumference at six months of age across pre- and post-vaccination periods.

Regarding comparing anthropometry between the pre and post-vaccination groups against COVID-19, weight at six months was higher in the pre-vaccination group (*p* < 0.01) but without a significant difference in the Z score of weight. Despite this, the pre-vaccination group presented higher length (*p* < 0.01) and Z score of length at six months (*p* < 0.01). Also, the pre-vaccination group had higher head circumference at six months (*p* < 0.01) but no difference in the Z score head circumference—Table 4 and Figure 1. At six months, the prevalence of eutrophy was higher in the post- vaccination group than in the pre-vaccination group (83.8% vs. 77.9%). The risk of overweight followed a similar pattern, slightly higher before vaccination (9.3% vs. 7.4%). On the other hand, being overweight was more common in the post-vaccination group (1.2% vs. 1.9%), while obesity was more frequent in the pre-vaccination group (3.5% vs. 0.5%). Regarding height at birth, short stature was notably more significant in the pre-vaccination group (22.1% vs. 11.1%); however, at six months, it was more prevalent in the post-vaccination group (12.8% vs. 18.1%). For head circumference, microcephaly was more common at birth pre-vaccination (17.2% vs. 5.6%), but at six months, it increased significantly in the post-vaccination group (7% vs. 10.2%) (data not presented in tables).

**Table 4:** Comparison of anthropometric evolution at birth and at 6 months of corrected gestational age between groups according to period of birth pre and post-vaccination against COVID-19.

	At birth			6 months		
	Pre-Vaccination	Post-Vaccination	*p*	Pre-Vaccination	Post-Vaccination	*p*
Gestational age (s)	32.8 ± 2.7	32.3 ± 2.8	0.16	64.3 ± 6.7	61.9 ± 6.7	<0.01*
Weight (g)	1.873.6 ± 614	1.732.3 ± 533.8	0.06	7.058.9 ± 1.381.7	6.582 ± 1.310.9	<0.01*
Z score of weight	−0.28 ± 1.02	−0.34 ± 0.93	0.57	−0.42 ± 1.44	−0.69 ± 1.19	0.09
Length (cm)	42.7 ± 4.3	40.8 ± 4.0	<0.01*	64.9 ± 4.7	62.8 ± 4.6	<0.01*
Z score of length	−0.20 ± 1.20	−0.79 ± 1.14	<0.01*	−0.25 ± 1.75	−0.82 ± 1.34	<0.01*
Head circujmference (cm)	29.4 ± 2.9	29.2 ± 2.9	0.68	42.4 ± 2.4	41.5 ± 3.6	<0.01*
Z score of head circumference	−0.44 ± 1.21	−0.28 ± 1.23	0.29	−0.00 ± 1.59	−0.32 ± 1.49	0.09
Anthropometric evolution						
Δ Z score of weight	–	–		1.09 ± 1.37	0.92 ± 1.06	0.24
Δ Z score of length	–	–		−0.05 ± 1.80	−0.03 ± 1.28	0.88
Δ Z score of head circumference	–	–		0.44 ± 1.61	−0.05 ± 1.46	0.01

Numerical variables presented as mean ± standard deviation; *n*, number; s, weeks; g, grams; Δ, variation; Student *t* test for numerical variables.

* Means statistically significant.

In the post-vaccination period, the analysis of variance revealed a statistical difference in the type of breastfeeding in Z scores for weight (*p* = 0.01), length (*p* = 0.01), and head circumference (*p* = 0.04). Infants submitted to artificial breastfeeding with added carbohydrates and mixed breastfeeding presented higher averages in Z scores of weight and length, in contrast of those fed exclusively with breast milk, presented higher mean Z scores of head circumference—Table 5.

**Table 5:** Analysis of variance of Z-scores for weight, length and head circumference by type of breastfeeding with period of birth before and after vaccination against COVID-19.

Variable	Group	Breastfeeding type	*n*	Average SD	*P* *
Z score 6 months					
Weight	Pre-Vaccine	Exclusive breastfeeding	11	−0.79 ± 1.06	0.31
		Mixed breastfeeding	25	−0.04 ± 1.33	
		Artificial breastfeeding	45	−0.59 ± 1.60	
		Artificial breastfeeding with added carbohydrates	5	0.03 ± 0.09	
	Post-Vaccine	Exclusive breastfeeding	30	−0.49 ± 1.10	0.01
		Mixed breastfeeding	64	−0.44 ± 0.95	
		Artificial breastfeeding	109	−0.96 ± 1.23	
		Artificial breastfeeding with added carbohydrates	12	−0.08 ± 0.95	
Length	Pre-Vaccine	Exclusive breastfeeding	11	−0.99 ± 1.36	0.45
		Mixed breastfeeding	25	−0.05 ± 1.30	
		Artificial breastfeeding	45	−0.24 ± 2.04	
		Artificial breastfeeding with added carbohydrates	5	0.24 ± 1.68	
	Post-Vaccine	Exclusive breastfeeding	30	−0.68 ± 1.22	0.01
		Mixed breastfeeding	64	−0.56 ± 1.19	
		Artificial breastfeeding	109	−1.11 ± 1.43	
		Artificial breastfeeding with added carbohydrates	12	−0.17 ± 0.98	
Head circumference	Pre-Vaccine	Exclusive breastfeeding	11	−0.16 ± 1.21	0.26
		Mixed breastfeeding	25	0.43 ± 1.13	
		Artificial breastfeeding	45	−0.27 ± 1.84	
		Artificial breastfeeding with added carbohydrates	5	0.61 ± 1.59	
	Post-Vaccine	Exclusive breastfeeding	30	0.07 ± 1.08	0.04
		Mixed breastfeeding	64	−0.06 ± 1.47	
		Artificial breastfeeding	109	−0.62 ± 1.59	
		Artificial breastfeeding with added carbohydrates	12	−0.13 ± 0.98	

*N*, number; SD, standard deviation.

* ANOVA.

In the linear regression analysis by groups, the Z score of weight at six months was negatively affected by congenital abnormalities both before (*β* = −0.358, *p* = 0.000) and after vaccination (*β* = −0.190, *p* = 0.030). Furthermore, in the post-vaccination period, EUGR (*β* = −0.407, *p* = 0.000) and anemia (*β* = −0.22, *p* = 0.014) contributed negatively. EUGR consistently reduced the Z score of length in both periods (*β* = −0.261, *p* = 0.011; *β* = −0.347, *p* = 0.000), with additional influences of congenital abnormalities (*β* = −0.266, *p* = 0.030) and a positive effect of the type of breastfeeding (*β* = 0.244, *p* = 0.012) in the pre-vaccination period. As for the Z score of head circumference, congenital abnormalities impaired its development in both the pre and post-vaccination groups (*β* = −0.209, *p* = 0.040; *β* = −0.187, *p* = 0.007), with additional influences negative for bronchopulmonary dysplasia (*β* = −0.485, *p* = 0.011) and positive for weight stratification at birth (*β* = 0.198, *p* = 0.042) in pre-vaccination, while post vaccination, maternal addictions (*β* = −0.250, *p* = 0.000) and parenteral nutrition (*β* = −0.199, *p* = 0.003) were negative factors (data not presented in tables).

## 4 Discussion

In the studied sample, there was a lower prevalence of moderate and late premature babies compared to data published by the WHO (1), which was associated with an increase in the prevalence of extremely premature babies in the sample, probably due to the socioeconomic characteristics of the sample municipality. Comorbidities most prevalent in participants was the use of parenteral nutrition, neonatal sepsis, and the presence of RDS, which increase the length of stay in the neonatal ICU and cause EUGR with adverse effects on growth and neurological development of premature babies in the long term (17).

The prevalence of microcephaly at birth, based on head circumference measurements, was observed in newborns with generally unfavorable conditions, such as prematurity and low socioeconomic status (18). At six months of corrected gestational age, the average head circumference and Z scores of head circumference indicates growth within acceptable ranges, with a reduction in the prevalence of microcephaly and an increase in macrocephaly. The head circumference growth is a critical indicator of neurological development in premature infants. The head circumference below the 10th percentile at birth and at discharge from the neonatal ICU are associated with lower scores in motor, cognitive, and language systems assessments at 4.5 years of age (19).

The growth of the premature newborn must follow the intrauterine growth pattern. However, the maintenance of growth rate is complex due to perinatal comorbidities, mainly related to weight at birth, gestational age, and severity of initial neonatal pathology. These comorbidities determine increased metabolic needs and release of catabolism-stimulating factors, with consequent EUGR and post-discharge anthropometric changes (20–22). In the sample studied, the EUGR had a negative impact in Z scores of weight, length and head circumference at six months. The main comorbidities associated with worse outcomes in anthropometric measurements were anemia, patent ductus arteriosus, low weight at birth, and congenital abnormalities. However, the presence of infections tends to have a positive effect on anthropometry, which possibly reflects an overdiagnosis of sepsis at birth, as clinical manifestations are often not sufficient to differentiate newborns with proven sepsis from newborns with unproven sepsis, resulting in overdiagnosis (23, 24).

Anthropometry at six months of corrected age shows an adequate weight gain, with most children entered the eutrophic range. Catch-up can be defined by a variation in the Z score greater than 0.67, corresponding to the rise of a channel in the percentile or standard deviation curves (25). A more considerable weight catch-up in the first years of life, exceeding the Z score established for head circumference and length, as in the analyzed sample, is associated with a greater risk of developing arterial hypertension, type II diabetes and cardiovascular disease in adolescence or young adulthood. This occurs due to structural and functional subclinical changes in target organs exposed to stressful events in the perinatal period, and suboptimal nutrition evidenced by the EUGR, associated with excessive catch-up of weight (26–29). Furthermore, 10.4% of the studied infants had high BMI for their age, warning of the potential risk of being overweight or child obesity.

On the other hand, the prevalence of short stature and the decrease in Z score of length at six months highlights the need for continuous monitoring of linear growth, which may reflect the delay in growth due to extreme prematurity. Premature babies may experience recovery in linear growth throughout the first years of life, but this often depends on continued care and adequate nutrition (29–31). Regarding the nutritional approach, premature newborns present an increase in demand nutritional due to the greater risk of comorbidities, and the best nutritional approach has yet to be defined despite technological advances. This approach provides adequate growth and development without increasing the risk of future diseases (32).

This study evaluated children before the start of vaccination against COVID-19 and after the start of immunization in Brazil to analyze the impacts of the vaccine on pregnant women and their premature newborns. Homogeneity was evident between the groups in most characteristics, but there was an increase in moderate and late preterm births due to a reduction in extreme prematurity in the post-vaccination group, which may reflect a positive impact of vaccination. According to data from Fiocruz and WHO, vaccination against COVID-19 significantly reduced gestational complications, even before the inclusion of pregnant women in the group's priorities and was demonstrated to be effective in reducing the incidence of ICU admissions, deaths, and neonatal complications, such as premature births, resulting from SARS-CoV-2 (6).

In the pre-vaccination group, there was a higher prevalence of LGA newborns in addition to weight, length, Z scores of length and head circumference greater at six months, reflecting the impacts of the period of social isolation on pregnant women with a decrease in the prevalence of extremely premature babies and increase in late and moderate prematurity, probably due to better conditions birth. Thus, the best anthropometric outcome reflects better conditions at birth, associated with the period of social isolation, with less exposure to postnatal infections. In the analysis of the pre and post-vaccine groups, the Z score of weight at six months was negatively affected by congenital abnormalities, both before and after vaccination. Although no difference was observed significant in the prevalence of congenital abnormalities between groups, congenital anomalies are configured as essential health problems that cause death in childhood, chronic diseases, and disabilities (33).

In the post-vaccination group, EUGR was more harmful on the Z score of weight, length, and head circumference at six months, probably due to the higher prevalence of prenatal complications in this group compared to the pre-vaccination group, in which the EUGR only impacted the Z score of length more mildly. This may be associated with a period of more significant maternal stress, as those born in the post-vaccination period had their mothers during the gestational period in isolation. The COVID-19 pandemic has affected the outcome of pregnancy, increasing morbidities and complications during pregnancy, childbirth, and postpartum. Furthermore, the extensive quarantine periods have disrupted the healthcare system and made access to healthcare services difficult for prenatal control (34).

Also, a higher prevalence of viral bronchiolitis and wheezing was evidenced in the post-vaccine group, which had an unfavorable impact on weight and head circumference at six months, probably due to greater flexibility in social isolation measures. According to Min Li et al. (2020), during the COVID-19 pandemic, the weight of term or close to term newborns was significantly higher in the group that remained in social isolation in comparison to the control group, which may indicate that intrauterine conditions for pregnancies may have been favorable despite the restrictions and stresses associated with isolation during this period.

Furthermore, the pre-vaccination group had a higher prevalence of gastroesophageal reflux. The episodes of regurgitation are more frequently observed in artificially breastfed infants than in breastfed infants (35). During the COVID-19 pandemic in several regions, there was a decline in breastfeeding exclusivity and an increase in early weaning associated with maternal work and uncertainty regarding guidelines health (7, 8, 36). Also, concern about the transmission of the SARS-CoV-2 virus contributed to a reduction in initiation or maintenance of breastfeeding despite continued evidence of breast milk as the ideal source of neonatal nutrition (9). With the start of vaccination, concerns about transmissibility may have decreased, an improvement in the prevalence of breastfeeding, and a drop in gastroesophageal reflux.

Among the study's limitations, data collection based on medical records may induce bias of information due to the variability in the accuracy of recording these data, which was minimized by the fact that the author completed all records of the work and was responsible for monitoring outpatient clinics for all newborns. The lack of detail on specific interventions received by premature babies in the neonatal ICU may have directly influenced the observed growth outcomes. Also, conclusions about differences between pre and post-vaccination groups may be limited, as the vaccination began in January 2021, and pregnant women were formally included in the priority groups in mid-2021 in Brazil.

Despite these limitations, the findings reinforce the need for robust health policies to protect this vulnerable population in times of global crisis, such as the COVID-19 pandemic. O continuous monitoring and nutritional support, combined with appropriate public policies, are crucial for the growth and development of premature babies.

## 5 Conclusion

During the COVID-19 pandemic, non-nutritional factors significantly influenced the anthropometry of premature babies. The EUGR, which may reflect prenatal conditions during the maternal social isolation period, was one of the foremost prevalent comorbidities and is a significant challenge as it impacts anthropometric outcomes after hospital discharge, requiring more effective therapeutic strategies. Therefore, public health measures must be planned to reduce complications in pregnancy and optimize the growth and development of premature infants.

## Data Availability

The raw data supporting the conclusions of this article will be made available by the authors, without undue reservation.

## References

[1] Chawanpaiboon S Vogel JP Moller AB Lumbiganon P Petzold M Hogan D Global, regional, and national estimates of levels of preterm birth in 2014: a systematic review and modelling analysis. Lancet Glob Health. (2019) 7(1):e37–46. 10.1016/S2214-109X(18)30451-0 30389451 PMC6293055

[2] Gurol-Urganci I Jardine JE Carroll F Draycott T Dunn G Fremeaux A Maternal and perinatal outcomes of pregnant women with SARS-CoV-2 infection at the time of birth in England: national cohort study. Am J Obstet Gynecol. (2021) 225(5):522.e1–522.e11. 10.1016/j.ajog.2021.05.016 34023315 PMC8135190

[3] Howson CP Kinney MV McDougall L Lawn JE, Born Too Soon Preterm Birth Action Group. Born too soon: preterm birth matters. Reprod Health. (2013) 10(Suppl 1):S1. 10.1186/1742-4755-10-S1-S1 24625113 PMC3828581

[4] Ramos HÂC Cuman RKN. Fatores de risco para prematuridade: pesquisa documental. Escola Anna Nery. (2009) 13(2):297–304. 10.1590/S1414-81452009000200009

[5] World Health Organization. Considerations for Quarantine of Individuals in the Context of Containment for Coronavirus Disease (COVID-19): Interim Guidance, 19 March 2020. Geneva: WHO (2020).

[6] Duque Estrada C Nóbrega L. COVID-19: *Balanço de Dois Anos da Pandemia Aponta Vacinação Como Prioridade*. Agência Fiocruz de Notícias. (2022). p. 1–29. Available online at: https://portal.fiocruz.br/sites/portal.fiocruz.br/files/documentos_2/boletim_covid_2022-balanco_2_anos_pandemia-redb.pdf (accessed November 28, 2024).

[7] Holand BL de Oliveira Agostini C Pacheco MCM de Leon DMZ Drehmer M Bosa VL. Association between breastfeeding and complementary feeding in pre-pandemic and pandemic COVID-19 times: maternar cohort study. J Pediatr. (2022) 98(5):496–503. 10.1016/j.jped.2021.12.007 PMC8801333 35139343

[8] Silva IPP Lopes IMD. Comparative study on breastfeeding in the context of the COVID-19 pandemic at a baby friendly hospital in Northeast Brazil. Res Soc Dev. (2022) 11(16):e50111637976. 10.33448/rsd-v11i16.37976

[9] Nyirongo M Agrawal N Rojas A Barbour K. Impact of the coronavirus disease (COVID-19) pandemic on neonatal nutrition: focus on low- and middle-income countries. Curr Trop Med Rep. (2022) 9:218–24. 10.1007/s40475-022-00272-7 36415223 PMC9672601

[10] UNICEF. Breastfeeding safely during the COVID-19 pandemic. (2023). Available online at: https://www.unicef.org/brazil/media/2641/file/Amamentar-com-seguranca-durante-a-pandemia-COVID-19.pdf (accessed April 01, 2024).

[11] WHO, Intergrowth 21-st. Implementing international postnatal growth standards for preterm infants. (2023). Available online at: https://www.gfmer.ch/omphi/interpractice/pdf/M1P.pdf (accessed April 01, 2024).

[12] Brasil. Ministério da Saúde. Secretaria de Atenção à Saúde. Departamento de Atenção Básica. Orientações Para a Coleta e Análise de Dados Antropométricos em Serviços de Saúde: Norma Técnica do Sistema de Vigilância Alimentar e Nutricional—SISVAN. Brasília: Ministério da Saúde; 2015.

[13] World Health Organization. WHO Child Growth Standards: Length/Height-for-Age, Weight-for-Age, Weight-for-Length, Weight-for-Height and Body Mass Index-for-Age: Methods and Development. Geneva: WHO (2006).

[14] Sociedade Brasileira de Pediatria (SBP). Dia Mundial da Prematuridade. (2019). Available online at: https://www.sbp.com.br/fileadmin/user_upload/DocCientNeonatol_SBP_Prematuridade_18112019__1_.pdf (Novembro 17, 2019).

[15] World Health Organization. Physical status: The use and Interpretation of Anthropometry. Report of a wHO Expert Committee. Geneva: WHO (1995). (Technical Report Series, 854). 8594834

[16] Brasil. Ministério da Saúde. Secretaria de Atenção à Saúde. Departamento de Atenção Básica. Saúde da criança: Aleitamento Materno e Alimentação Complementar. 2ª ed. Brasília: Ministério da Saúde; 2015.

[17] Martínez-Jiménez MD Gómez-García FJ Gil-Campos M Pérez-Navero JL. Comorbidities in childhood associated with extrauterine growth restriction in preterm infants: a scoping review. Eur J Pediatr. (2020) 179(8):1255–65. 10.1007/s00431-020-03613-8 32096070

[18] Marinho F Araújo V Porto D Ferreira H Coelho M Lecca R Microcefalia no Brasil: prevalência e caracterização dos casos a partir do sistema de informações sobre nascidos vivos (sinasc), 2000–2015. Epidemiol Serv Saúde. (2016) 25(4):701–12. 10.5123/S1679-49742016000400004 27869983

[19] Selvanathan T Guo T Kwan E Chau V Brant R Synnes A Head circumference, total cerebral volume and neurodevelopment in preterm neonates. Arch Dis Child Fetal Neonatal Ed. (2022) 107:181–7. 10.1136/archdischild-2020-321397 34261769

[20] Hay WW Jr. Strategies for feeding the preterm infant. Neonatology. (2008) 94(4):245–54. 10.1159/000151643 18836284 PMC2912291

[21] Jasper EA Cho H Breheny PJ Bao W Dagle JM Ryckman KK. Perinatal determinants of growth trajectories in children born preterm. PLoS One. (2021) 16(1):e0245387. 10.1371/journal.pone.0245387 33507964 PMC7842887

[22] Silveira RC Procianoy RS. Padrões de crescimento pós-natal do recém-nascido prematuro: como avaliar. J Pediatr. (2019) 95(Suppl 1):S42–8. Available online at: doi:http://dx.doi.org/10.1016/j.jped.2018.10.006. 10.1016/j.jped.2018.10.006

[23] Huetz N Launay E Gascoin G Leboucher B Savagner C Muller JB Potential impact of umbilical-cord-blood procalcitonin-based algorithm on antibiotics exposure in neonates with suspected early-onset sepsis. Front Pediatr. (2020) 8:127. 10.3389/fped.2020.00127 32363168 PMC7181674

[24] Puopolo KM Benitz WE Zaoutis TE, Committee on Fetus and Newborn; Committee on Infectious Diseases. Management of neonates born at ≥35 0/7 weeks’ gestation with suspected or proven early-onset bacterial sepsis. Pediatrics. (2018) 142(6):e20182894. 10.1542/peds.2018-2894 30455342

[25] Ong KK Ahmed ML Emmett PM Preece MA Dunger DB. Association between postnatal catch-up growth and obesity in childhood: prospective cohort study. Br Med J. (2000) 320(7240):967–71. 10.1136/bmj.320.7240.967 10753147 PMC27335

[26] Chehade H Simeoni U Guignard JP Boubred F. Preterm birth: long term cardiovascular and renal consequences. Curr Pediatr Rev. (2018) 14(4):219–26. 10.2174/1573396314666180813121652 30101715 PMC6416185

[27] Martin RA Walsh M. Fanaroff and Martin's Neonatal-Perinatal Medicine. 11th ed. 2-volume set. Philadelphia: Elsevier (2023).

[28] Rugolo LM. Crescimento e desenvolvimento a longo prazo do prematuro extremo. J Pediatr (Rio J). (2005) 81(1 Suppl):101–10. 10.2223/1309 15809691

[29] Uhing MR Das UG. Optimizing growth in the preterm infant. Clin Perinatol. (2009) 36(1):165–76. 10.1016/j.clp.2008.09.010 19161873

[30] Ong KK Kennedy K Castañeda-Gutiérrez E Forsyth S Godfrey K Koletzko B Postnatal growth in preterm infants and later health outcomes: a systematic review. Acta Paediatr. (2015) 104(10):974–86. 10.1111/apa.13128 26179961 PMC5054880

[31] Villar J Giuliani F Barros F Roggero P Zarco I Rego MA Monitoring the postnatal growth of preterm infants: a paradigm change. Pediatrics. (2018) 141(2):e20172467. 10.1542/peds.2017-2467 29301912

[32] American Academy of Pediatrics Nutrition. Nutritional Needs of the Preterm Infant. (2022).

[33] World Health Organization. Congenital anomalies. (2016). Available online at: https://www.who.int/en/newsroom/fact-sheets/detail/congenital-anomalies (accessed April 01, 2024).

[34] Li M Yin H Jin Z Zhang H Leng B Luo Y Impacto do lockdown de Wuhan nas indicações de parto cesáreo e Ps dos RNs durante o período epidêmico de COVID-19. PLoS One. (2020) 15(8):e0237420. 10.1371/journal.pone.0237420 32790709 PMC7425855

[35] Giovanni C Cavaliere M D’Eufemia P Pelliccia A Celli M Porcelli M Sandifer’s syndrome in a breast-fed infant. Am J Perinatol. (2000) 17(3):147–50. 10.1055/s-2000-9285 11012139

[36] Duque Estrada C Nóbrega L. Covid-19: Balanço de Dois Anos da Pandemia Aponta Vacinação Como Prioridade. Agência Fiocruz de Notícias (2022). Available online at: https://fiocruz.br.

